# Predicting Essential Proteins Based on Integration of Local Fuzzy Fractal Dimension and Subcellular Location Information

**DOI:** 10.3390/genes13020173

**Published:** 2022-01-19

**Authors:** Li Shen, Jian Zhang, Fang Wang, Kai Liu

**Affiliations:** 1 College of Agronomy, Hunan Agricultural University, Changsha 410128, China; li_shen2000@sina.com; 2 College of Information and Intelligence, Hunan Agricultural University, Changsha 410128, China; gustlk@163.com

**Keywords:** essential proteins, PPI network, LFFD, subcellular location information

## Abstract

Essential proteins are indispensable to cells’ survival and development. Prediction and analysis of essential proteins are crucial for uncovering the mechanisms of cells. With the help of computer science and high-throughput technologies, forecasting essential proteins by protein–protein interaction (PPI) networks has become more efficient than traditional approaches (expensive experimental methods are generally used). Many computational algorithms were employed to predict the essential proteins; however, they have various restrictions. To improve the prediction accuracy, by introducing the Local Fuzzy Fractal Dimension (LFFD) of complex networks into the analysis of the PPI network, we propose a novel algorithm named LDS, which combines the LFFD of the PPI network with the protein subcellular location information. By testing the proposed LDS algorithm on three different yeast PPI networks, the experimental results show that LDS outperforms some state-of-the-art essential protein-prediction techniques.

## 1. Introduction

As one of the important gene products, proteins play a critical role in the lifespan of cells for all living organisms. Essential proteins are those that cause lethality or infertility of a cell if only one of them is removed [1]. Organisms cannot survive without essential proteins [2,3]. Therefore, the prediction of essential proteins is a meaningful task due to its theoretical interest and practical significance.

Up to now, there are generally two kinds of methods used to predict essential proteins. One is the traditional biological experimental techniques, such as gene knockouts [4], RNA interference [5], and conditional knockouts [6]. All of them are expensive and time-consuming. Another is the computational approaches with the advantage of efficient and low-cost owing to high throughput technologies, such as mass spectrometry analysis [7], yeast two-hybrid system [8,9], and tandem affinity purification [10]. Many computational approaches have been proposed from the network perspective to capture the relations between network features and protein essentiality. If each protein is regarded as a node, the protein–protein interaction (PPI) network can be understood by the concept of a complex network. Complex network-related methods have long been used in PPI networks studies [11,12,13,14,15].

In the current study of the PPI networks, an interesting finding uncovers that highly connected proteins are more likely to be essential ones. This is called the centrality–lethality rule. Accordingly, more and more research efforts focus on the correlations between PPI network topological centrality and protein essentiality. Among them, a wealth of methods have emerged, such as Degree Centrality (DC) [16,17], Subgraph Centrality (SC) [18], Betweenness Centrality (BC) [19], Closeness Centrality (CloseC) [20], Clustering Coefficient (ClusterC) [21], and Information Centrality (IC) [22]. Li et al. [23] proposed a local average connectivity (LAC) to identify essential proteins. Qi et al. [24] utilized the local interaction density (LID) of the PPI network to predict essential proteins. The above methods provide a new idea for predicting essential proteins. However, due to the high proportion of false positives and false negatives in the PPI networks, they also have certain shortcomings. Taking account of the defect of PPI networks, biological information of proteins should also be considered, including protein complex information, gene expression data, orthologous protein information, subcellular localization information, and so on. Li et al. [25] developed a PeC method that integrates PPI information (edge clustering coefficient) and gene expression profiles (Pearson’s correlation coefficient of two interacting proteins) for discovery of essential proteins. Lei et al. [26] designed a weighted PPI network by applying Hyperlink-Induced Top Search (HITS) for essential proteins mining. Ren et al. [27] predicted essential proteins by incorporating PPI networks and protein-complex information. Because essential proteins are usually interconnected, Peng et al. [28] introduced an iterative method for identifying essential proteins based on orthology and PPI networks. Recently, plenty of research has demonstrated that subcellular localization plays a key role in predicting essential protein. Accordingly, Tang et al. [29] proposed a new method by combing the subcellular localization information and PPI data. The experimental results show that it raises the recognition accuracy of essential proteins.

In Ref. [30], Song et al. reported that PPI networks are a fractal network and therefore possesses topological self-similarity [31]. This provides a theoretical basis for predicting essential proteins according to the fractal dimension of the PPI network. A large number of fractal dimension algorithms have been put forward, for instance, box-covering algorithm [32], ball-covering algorithm [33], and edge-covering box-counting algorithm [34], to be used to analyze various complex networks in the real world. However, the algorithms mentioned are all aimed at the global fractal structure of complex networks but ignore the characterization of every node. To make up for this defect, Filipi et al. [35] proposed the local fractal dimension (LFD) of complex networks and apply it to analyze two power grid networks. They found that nodes with high LFD are mostly the topological center of networks.

In this paper, we first develop a new LFD combing with an idea of the fuzzy set, which is called the local fuzzy fractal dimension (LFFD). Compared with the LFD, the LFFD can accurately reflect the role of nodes in the networks. Next, we obtain the subcellular location information of essential and non-essential proteins of Saccharomyces cerevisiae. Then, the subcellular compartment score can be determined using the Bayes formula. Next, combining the LFFD and the subcellular compartment score, we present a so-called LDS algorithm to predict the essential proteins. Three PPI datasets are employed to test our algorithm. On the same datasets, nine existing methods are used for comparison. The result shows that LDS brings the best result.

## 2. Methods

### 2.1. Local Fractal Dimension

A protein–protein interaction (PPI) network is generally denoted as an undirected network *G* = (*V*, *E*), which is composed of node set *V* and an edge set *E*. Each node *v* ∈ *V* represents a protein, each edge (*u*, *v*) ∈ *E* represents an interaction between protein *u* and protein *v*.

It is widely known that most real-world networks obey the power-law distribution. In Ref. [31], the authors show that the distribution of the PPI network is also according to the power law. According to the power law, Equation (1) holds for the PPI network,

(1) $$B_{v}(r)=Cr^{D_{v}}$$

where *B_v_*(*r*) is the total number of nodes in the sphere (including the boundary) with center node *v* and topological radius *r*. *r* is taken from 1 to the farthest distance from node *v* to others. *D_v_* is the local fractal dimension (LFD) of node *v*, and *C* is constant. The fractal dimension *D_v_* can be calculated by the derivatives between the logarithm of *B_v_*(*r*) and *r*, as follows. In general, one can obtain the *D_v_* by calculating the fitting slope of the straight line in the double-log of *B_v_*(*r*) and *r*.

(2) $$D_{v}=\frac{d}{d\ln r}\ln B_{v}(r)$$

**Figure 1:**
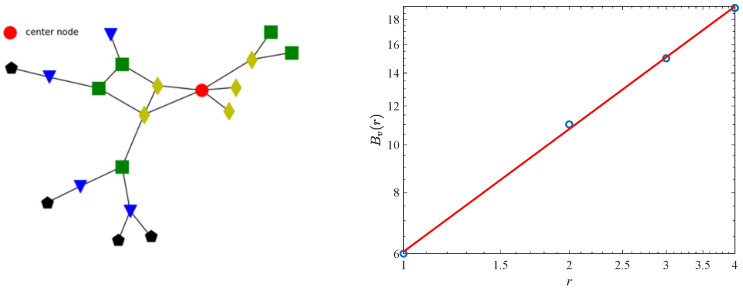
A simple example of calculating a local fractal dimension. The left is the network structure diagram. The right is the double-log plot between the *B_v_*(*r*) and *r*.

To visualize this process, we give an example as shown in Figure 1. The center node (red circle) is *v*, from *v* to the nodes with *r* = 1 (dark yellow diamond) and thus *B_v_* (1) = 6 (=1 + 5); from *v* to the nodes with *r* = 2 (green rectangular) and thus *B_v_* (2) = 11 (=6 + 5); from *v* to the nodes with *r* = 3 (blue triangle) and thus *B_v_* (3) = 15 (=11 + 4); and from *v* to the nodes with *r* = 4 (black pentagon) and thus *B_v_* (4) = 19 (=15 + 4). As calculated by Equation (2), the value of *D_v_* is 0.8295.

### 2.2. Local Fuzzy Fractal Dimension

In the calculation of the local fractal dimension, the nodes with a topological distance equal to or less than *r* are considered equally important. However, the distribution of these nodes is usually different and should not be treated equally. The closer to the center node, the greater the contribution to the center node. By this token, the local fractal dimension *D_v_* cannot truly describe the self-similarity of the PPI network. Here, we propose a method to calculate local fuzzy fractal dimension (LFFD) inspired by the concept of fuzzy set. In this method, the Gaussian membership function is employed to distinguish the contribution of different nodes to the center node. The LFFD is defined as

(3) $$D_{f}(v)=\frac{d}{d\ln r}\ln N_{v}(r)$$

where *D_f_*(*v*) denotes the LFFD of node *v*, *N_v_*(*r*) is the fuzzy value of the center node *v* and *r* is the topological radius. They are determined by

(4) $$A_{vj}(r)=e^{-\frac{d_{vj}^{2}}{2r^{2}}}$$

(5) $$N_{v}(r)=\frac{\sum A_{vj}(r)}{N}$$

where *d_vj_* is the shortest distance between node *v* and node *j*, *A_vj_* (*r*) is the Gaussian membership function value when *d_vj_* is less than or equal to *r*, and *N* is the total number of nodes whose shortest distance to the central node *v* is less than or equal to *r.* Taking *r* from 1 to the farthest distance from node *v* to others in the PPI network, the corresponding *N_v_*(*r*) is determined by averaging the membership value over the *N* nodes. Like the calculation process of *D_v_*, *D_f_*(*v*) can be calculated by the fitting slope of the straight line in the log-log plot between the *N_v_*(*r*) and *r*.

**Figure 2:**
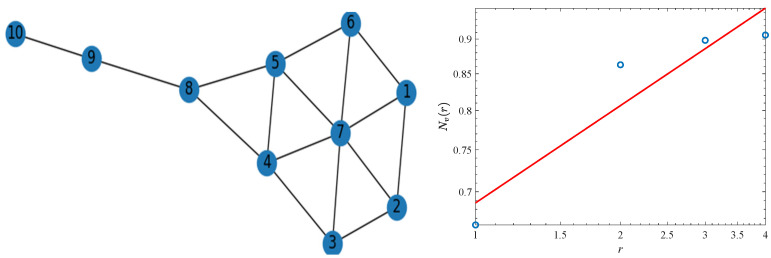
An example of calculating LFFD. The left is the kite network structure diagram. The right is the double-log plot between the *N_v_*(*r*) and *r*.

To show this method clearer, we take a well-known kite network as an example. In Figure 2, node 7 is the selected central node, and *r* is 1 to 4. The calculation of *N_v_*(*r*) is shown as follows.

$$N_{7}(1)=\frac{1}{7}\sum_{i=1}^{7}e^{-\frac{d_{i7}^{2}}{2\times 1}}=\frac{1}{7}\left(6e^{-\frac{1}{2}}+e^{0}\right)=0.6627$$

$$N_{7}(2)=\frac{1}{8}\sum_{i=1}^{8}e^{-\frac{d_{i7}^{2}}{2\times 4}}=\frac{1}{8}\left(6e^{-\frac{1}{8}}+e^{0}+e^{-\frac{1}{2}}\right)=0.8627$$

$$N_{7}(3)=\frac{1}{9}\sum_{i=1}^{9}e^{-\frac{d_{i7}^{2}}{2\times 9}}=\frac{1}{9}\left(6e^{-\frac{1}{2\times 9}}+e^{0}+e^{-\frac{4}{2\times 9}}+e^{-\frac{9}{2\times 9}}\right)=0.8981$$

$$N_{7}(4)=\frac{1}{10}\sum_{i=1}^{10}e^{-\frac{d_{i7}^{2}}{2\times 16}}=\frac{1}{10}\left(6e^{-\frac{1}{2\times 16}}+e^{0}+e^{-\frac{4}{2\times 16}}+e^{-\frac{9}{2\times 16}}+e^{-\frac{16}{2\times 16}}\right)=0.9059$$

Therefore, according to Equation (3), the LFFD of node 7 is 0.2312.

### 2.3. Subcellular Compartment Score

The scholars point out that subcellular location information has been widely exploited in the prediction of essential proteins [36]. We download the subcellular location data of Saccharomyces cerevisiae from the COMPARTMENTS database [37], which is classified into 11 different subcellular compartments, namely Cytoskeleton, Cytosol, Endoplasmic Reticulum, Endosome, Extracellular space, Golgi apparatus, Mitochondrion, Nucleus, Peroxisome, Plasma membrane, and Vacuole. By collecting from MIPS [38], SGD [39], DEG [40], and SGDP, we obtain a list of known 1285 essential proteins and 4394 non-essential proteins of Saccharomyces cerevisiae.

By analyzing the subcellular location data of identified essential and non-essential proteins, we develop a new evaluation strategy to obtain the subcellular compartment score, which is the probability that proteins in a subcellular compartment are potentially essential proteins. Firstly, we calculate the probability that the protein appears at each subcellular compartment in all 5679 (=1285 + 4394) protein data, which is defined as follows:

(6) $$P(C_{i})=P(E)P(C_{i}|E)+P(NE)P(C_{i}|NE)$$

where *C_i_* is the subcellular compartment with *i* from 0 to 10 and *P*(*C_i_*) is the probability that protein appears at *C_i_*. *P*(*E*) is the probability of essential proteins in 5679 proteins data, and *P*(*C_i_*|*E*) is the conditional probability, which indicates the probability that protein appears at *C_i_* in 1285 essential proteins. *P*(*NE*) is the probability of non-essential proteins in 5679 protein data, and *P*(*C_i_*|*NE*) indicates the probability that protein appears at *C_i_* in 4394 non-essential proteins. Then, the Bayes formula is employed to obtain the subcellular compartment score,

(7) $$P(E|C_{i})=\frac{P(E)P(C_{i}|E)}{P(C_{i})}$$

where *P*(*E*|*C_i_*) is the score of compartment *C_i_*, indicating the probability that the protein appearing at *C_i_* is an essential protein. According to the above method, the score of 11 subcellular compartments can be calculated. Finally, we count the subcellular compartment score of each protein in the PPI network. For some proteins, we compute the average value in the case of their subcellular location information containing multiple compartments, which is determined by

(8) $$SCS(v)=\frac{1}{N}\sum_{v\in C_{i}}P(E|C_{i})$$

where *N* is the subcellular compartment number of node *v*. *SCS*(*v*) is the final subcellular compartment score of node *v*. *SCS*(*v*) is set to 0 when the subcellular compartment of node *v* is null.

### 2.4. LDS Algorithm

The local fuzzy fractal dimension describes the topological feature of the PPI network, while the subcellular location information characterizes the biological information of the PPI network. To comprehensively assess the essentiality of every protein, we combine the above two characteristics to acquire the final value of each protein by using the LDS algorithm. The final value of protein *v* is defined as *LDS*(*v*), which is defined by

(9) $$LDS(v)=\alpha \times ND_{f}(v)+(1-\alpha )\times SCS(v)$$

where *ND_f_*(*v*) is the Min-Max normalization result of *D_f_*(*v*), and *α* is the parameter within the range (0, 1). If *α* is equal to 1, the *LDS*(*v*) only depends on the topological feature, and the *LDS*(*v*) is only determined by the biological information in the case of *α =* 0. All proteins in the PPI network are ranked in descending order of LDS value.

## 3. Results and Discussion

### 3.1. Experimental Data

As mentioned above, the PPI network of Saccharomyces cerevisiae (yeast) has been widely used in studying essential proteins. In this work, we also use it to perform our experiment. Our PPI datasets were downloaded from the DIP database [41] and the MIPS database. After removing self-interactions and repeated interactions, we constructed three PPI datasets. They are the first dataset DIP4746 with 4746 proteins and 15,166 interactions from the DIP database, the second dataset DIP5093 with 5093 proteins and 24,743 interactions from the DIP database, and the third dataset MIPS4546 with 4546 proteins and 12,319 interactions from the MIPS database, respectively. In addition, we queried the essential and non-essential proteins and subcellular location information in each dataset. For the sake of discussion, we include the unknown proteins as non-essential proteins. More details are listed in Table 1.

### 3.2. Performance of the LDS Algorithm

**Figure 3:**
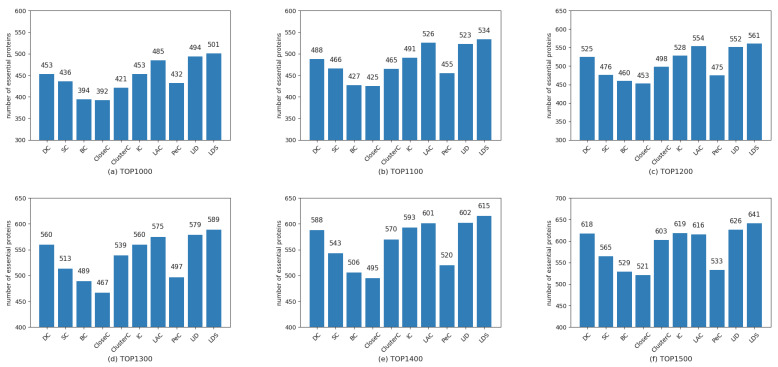
Comparison of the number of essential proteins predicted by LDS and other methods for dataset DIP4746. (**a**–**f**) are for the top 1000~1500, respectively.

**Figure 4:**
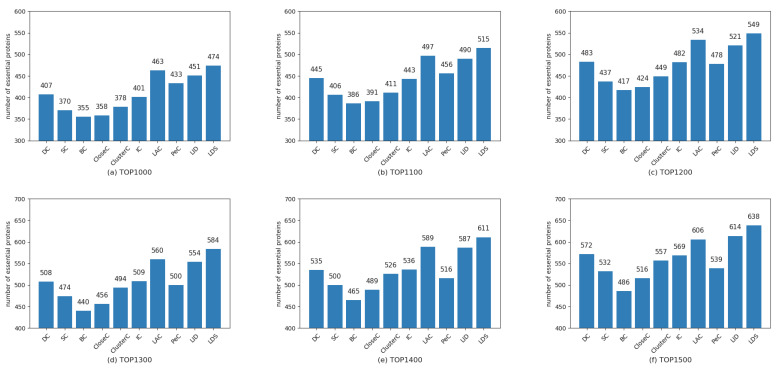
Comparison of the number of essential proteins predicted by LDS and other methods for dataset DIP5093. (**a**–**f**) are for the top 1000~1500, respectively.

**Figure 5:**
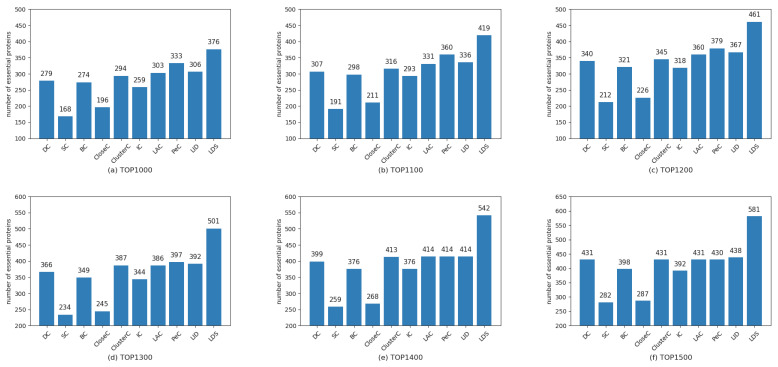
Comparison of the number of essential proteins predicted by LDS and other methods for dataset MIPS4546. (**a**–**f**) are for the top 1000~1500, respectively.

To demonstrate the performance of the LDS algorithm, we selected the top1000 to top1500 with step size 100 as the essential candidates by ranking proteins in descending order of the LDS value. Then, we checked the candidates with the collection of essential proteins mentioned in Section 2.3. As a comparison, the results obtained from the LDS and other nine traditional prediction methods, namely, DC, SC, BC, CloseC, ClusterC, IC, LAC, PeC, and LID, are shown in Figure 3, Figure 4 and Figure 5, respectively.

From these figures, some findings can be concluded: (1) The nine compared methods show different performance for the different datasets. For example, the methods LAC and LID outperform other methods on the datasets DIP4746 and DIP5093; however, they have mediocre performance on the dataset MIPS4546. The method PeC has the upper hand on the dataset MIPS4546 but is inferior to most methods over the former two datasets. The performance of the proposed LDS algorithm is quite stable. It showed the best performance for the three considered datasets. (2) Our proposed LDS algorithm performs slightly better for the dataset DIP4746 compared to other methods but is better than the others on the latter two datasets, especially for dataset MIPS4546. These findings suggest that the LDS is more suitable to predict essential proteins due to its high accuracy and robustness.

To further evaluate the performance of the proposed LDS algorithm comprehensively, six evaluation indexes, namely sensitivity (*SN*), specificity (*SP*), positive predictive value (*PPV*), negative predictive value (*NPV*), *F*-measure, and accuracy (*ACC*) are adopted here, defined as in Equations (10)–(15):

(10) $$SN=\frac{TP}{TP+FN}$$

(11) $$SP=\frac{TN}{TN+FP}$$

(12) $$PPV=\frac{TP}{TP+FP}$$

(13) $$NPV=\frac{TN}{TN+FN}$$

(14) $$F-measure=\frac{2\times SN\times PPV}{SN+PPV}$$

(15) $$ACC=\frac{TP+TN}{TP+TN+FP+FN}$$

where *TP* is the number of essential proteins correctly predicted as essential proteins and *TN* is the number of non-essential proteins correctly predicted as non-essential proteins. *FP* is the number of non-essential proteins incorrectly predicted as essential proteins, and *FN* is the number of essential proteins incorrectly predicted as non-essential proteins.

To assess the effectiveness of the LDS algorithm and the other methods, we select the top1500 of ranking results as essential proteins candidate set while the rest are categorized as non-essential proteins candidate set. The compared results calculated by using the LDS algorithm and the other nine methods on the three datasets are listed in Table 2. We highlight the best result for each dataset. As expected, all the highlighted results come from the LDS algorithm. It is confirmed again that LDS has a distinct advantage over other methods.

### 3.3. Influence of the Parameter α

**Figure 6:**
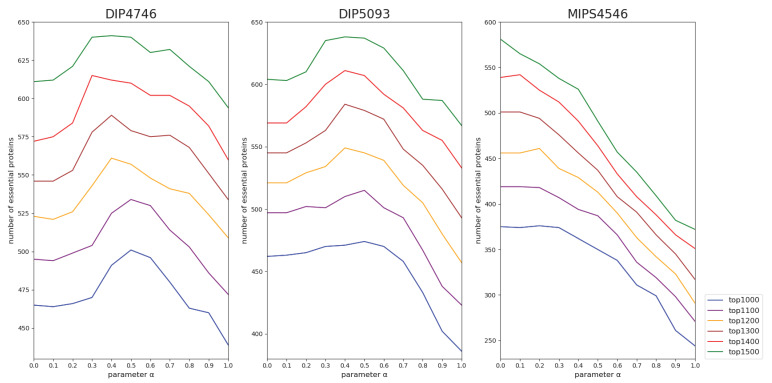
Number of essential proteins predicted by LDS in top1000–1500 for three datasets with different parameter *α*.

As shown in Equation (9), the parameter *α* (∈[0, 1]) is a weight value in the proposed LDS algorithm, which is used to balance the topological structure and biological information. Larger *α* means that the weight of fractal structure is greater. To illustrate how the *α* affects the result in the prediction of essential proteins, we changed the *α* in the range of [0, 1] with step size of 0.1 and redo our experiment reported in Section 3.2. The results are shown in Figure 6. We find that the prediction results depend greatly on *α*. Specifically, for the datasets DIP4746 and DIP5093, the best results are obtained from *α* taking 0.4~0.5, which suggests that both topological features and biological information are almost equally important for predicting the essential proteins in those two datasets. However, for the dataset MIPS4546, the optimum α that brings the best result is on the platform of 0~0.2, indicating that biological information is the main factor affecting the prediction of essential proteins. A potential reason for the difference of parameter values may be that Saccharomyces cerevisiae (yeast) datasets downloaded from different protein database websites have distinct topological features.

## 4. Conclusions

The prediction of essential proteins is an effective way to reveal the molecular mechanisms of cellular life. Based on the combination of the topological feature and biological information of the PPI network, we developed a novel LDS algorithm to predict essential proteins in this research. To investigate the performance of our proposed algorithm, we carried out several experiments on the three PPI datasets. The experiment results on the three datasets of Saccharomyces cerevisiae confirm that the LDS outperforms the other nine existing methods, namely DC, SC, BC, CloseC, ClusterC, IC, LAC, PeC, and LID. Six statistical indicators verify its advantage comprehensively.

In summary, this work is a primary attempt of the leading fractal nature of PPI to the prediction of essential proteins. The results suggest that it is significant to predict essential proteins by feature fusion. In a future study, we will focus on how to merge different features to improve prediction accuracy.

## Figures and Tables

**Table 1:** The information of the experimental data.

Datasets	Proteins	Interactions	Essential Proteins	Non-Essential Proteins
DIP4746	4746	15,166	1130	3616
DIP5093	5093	24,743	1167	3926
MIPS4546	4546	12,319	1016	3530

**Table 2:** Comparisons of *SN*, *SP*, *PPV*, *NPV*, *F*-measure, and *ACC* between LDS with other methods for three different PPI datasets. The bold is the best result.

Datasets	Methods	*SN*	*SP*	*PPV*	*NPV*	*F-Measure*	*ACC*
DIP4746	DC	0.5469	0.7561	0.412	0.8423	0.470	0.7063
	SC	0.500	0.7414	0.3767	0.8259	0.4297	0.6839
	BC	0.4681	0.7315	0.3527	0.8148	0.4023	0.6688
	CloseC	0.4611	0.7293	0.3473	0.8124	0.3962	0.6654
	ClusterC	0.5336	0.7519	0.402	0.8376	0.4586	0.700
	IC	0.5478	0.7564	0.4127	0.8426	0.4707	0.7067
	LAC	0.5451	0.7555	0.4107	0.8417	0.4684	0.7054
	PeC	0.4717	0.7326	0.3553	0.8161	0.4053	0.6705
	LID	0.554	0.7583	0.4173	0.8447	0.4760	0.7097
	**LDS**	**0.5673**	**0.7624**	**0.4273**	**0.8494**	**0.4875**	**0.716**
DIP5093	DC	0.4901	0.7636	0.3813	0.8344	0.4289	0.701
	SC	0.4559	0.7534	0.3547	0.8233	0.399	0.6853
	BC	0.4165	0.7417	0.324	0.8105	0.3645	0.6672
	CloseC	0.4422	0.7494	0.344	0.8188	0.387	0.679
	ClusterC	0.4773	0.7598	0.3713	0.8302	0.4177	0.6951
	IC	0.4876	0.7629	0.3793	0.8336	0.4267	0.6998
	LAC	0.5193	0.7723	0.404	0.8439	0.4544	0.7143
	PeC	0.4619	0.7552	0.3593	0.8252	0.4042	0.688
	LID	0.5261	0.7743	0.4093	0.8461	0.4604	0.7175
	**LDS**	**0.5467**	**0.7804**	**0.4253**	**0.8528**	**0.4784**	**0.7269**
MIPS4546	DC	0.4242	0.6972	0.2873	0.8079	0.3426	0.6362
	SC	0.2776	0.655	0.188	0.759	0.2242	0.5706
	BC	0.3917	0.6878	0.2653	0.7971	0.3164	0.6216
	CloseC	0.2825	0.6564	0.1913	0.7607	0.2281	0.5728
	ClusterC	0.4242	0.6972	0.2873	0.8079	0.3426	0.6361
	IC	0.3858	0.6861	0.2613	0.7951	0.3116	0.619
	LAC	0.4242	0.6972	0.2873	0.8079	0.3426	0.6362
	PeC	0.4232	0.6969	0.2867	0.8076	0.3418	0.6357
	LID	0.4311	0.6992	0.292	0.8102	0.3482	0.6392
	**LDS**	**0.5719**	**0.7397**	**0.3873**	**0.8572**	**0.4618**	**0.7022**

## Data Availability

The DIP dataset is available at https://dip.doe-mbi.ucla.edu/dip/Main.cgi. The MIPS dataset is available in http://mips.gsf.de.
